# Vaccination Programs for Adults in Europe, 2019

**DOI:** 10.3390/vaccines8010034

**Published:** 2020-01-20

**Authors:** Dimitrios C. Cassimos, Evgnosia Effraimidou, Snezana Medic, Theoharis Konstantinidis, Maria Theodoridou, Helena C. Maltezou

**Affiliations:** 1 Department of Pediatrics, Democritus University of Thrace, 68100 Alexandroupolis, Greece; dkasimos@med.duth.gr (D.C.C.); effraimidou@gmail.com (E.E.); 2 Department of Epidemiology, Faculty of Medicine, University of Novi Sad, 21000 Novi Sad, Serbia; snezana.medic@mf.uns.ac.rs; 3 Center for Disease Control and Prevention, Institute of Public Health of Vojvodina, 21000 Novi Sad, Serbia; 4 Blood Transfusion Center, University General Hospital of Alexandroupolis, 68100 Alexandroupolis, Greece; tkonsta@med.duth.gr; 5 Aghia Sophia Children’s Hospital, First Department of Pediatrics, University of Athens, 11527 Athens, Greece; 6 Department for Interventions in Health Care Facilities, National Organization of Public Health, 15123 Athens, Greece

**Keywords:** vaccinations, immunizations, programs, adults, Europe

## Abstract

Background: While all European countries implement vaccination programs for children, there are gaps in terms of vaccination programs for adults. Methods: We studied the 2019 vaccination policies for adults in 42 European countries. Results: Vaccination programs for adults were in place in all countries. However, there were considerable differences between countries in terms of number of vaccinations, target populations and frame of implementation (recommended or mandatory vaccinations). In particular the following vaccination policies were in place: influenza (42 countries), tetanus (31), diphtheria (30), pneumococcus (29), hepatitis B (20), pertussis (18), measles (14), human papilloma virus (14), meningococcus tetravalent A,C,W,Y (14), rubella (13), hepatitis A (11), mumps (11), poliomyelitis (10), herpes zoster (9), varicella (8), tick-born encephalitis (8), meningococcus B (6), rabies (6), *Haemophilus influenzae* type b (5), tuberculosis (3), typhoid fever (3), meningococcus C (2), and yellow fever (1). Seventeen countries implement mandatory vaccinations, mainly against diphtheria, tetanus and hepatitis B. Conclusions: There are significant differences in vaccination programs for adults in Europe. Routine vaccination programs for adults need to be strengthened. A consensus-based vaccination program is needed.

## 1. Introduction

The wide implementation of vaccination programs during childhood in the second half of the 20th century constituted one of the most successful public health policies in human history, leading to the eradication or control of several fearful diseases of the past [1,2]. However, despite the long-standing vaccination programs, large epidemics of measles, mumps, rubella, pertussis and hepatitis A occurred in Europe in the past decade [3,4,5,6]. In these epidemics young adults were disproportionately affected with thousands of notified cases and tens of fatalities [3,4,5,6]. The suboptimal vaccination coverage rates among children and adults, for various reasons, but also the gaps on vaccination policies and schemes specifically for adults in several countries provided the appropriate background for these epidemics. For Europe, as for other modern societies, current challenges in terms of vaccine policies, include vaccine hesitancy (the World Health Organization recognized vaccine hesitancy as one of the ten threats for global health in 2019) [7], the large influx of migrants and refugees, but also issues of accessibility to vaccine services, mandatory vaccination policies, new vaccines and new indications for vaccinations [8,9,10,11,12,13,14,15]. 

The modern public health policies on vaccines rely on the transition from ‘pediatric vaccination programs’ to ‘vaccination programs for all ages and all groups’. The rationale for vaccinating adults aims for providing additional protection against: first, waning immunity (e.g., encountered especially among adolescents or young adults following natural pertussis disease or pertussis vaccination); second, increased morbidity and mortality of several vaccine preventable diseases (VPDs) during adulthood (e.g., measles, varicella, hepatitis A); third, age-related dysregulation and decline of the immune system in older adults-collectively termed ‘immunosenescence’; and fourth, co-morbidities associated with ageing [6,14,16,17,18]. The increase of life expectancy in Europe renders older people an important proportion of the general population and healthy ageing is being increasingly incorporated in modern healthcare systems [18]. In 2019 persons ≥65 years old accounted for approximately 20% of the European population, and this is expected to increase to approximately 30% by 2050 [19].

While all European countries implement routine vaccination programs for children for several decades, there are gaps in terms of vaccination programs for adults. A 2010 review of vaccination programs for adults showed that only 12 of 31 developed countries (38.7%) had comprehensive adult vaccination schedules, with significant variations in terms of number of vaccines or vaccine components [20]. In this article we studied the current national vaccination programs for adults in 42 European countries.

## 2. Methods

We searched official governmental and national public health websites for information on routine vaccination programs for adults in European countries in effect in 2019. We also searched the websites of the European Centre for Disease Prevention and Control (ECDC) and the World Health Organization (WHO) [21,22]. Data from the various official sources were compared and analyzed in order to establish reliable information on vaccine policies in place in each country. The websites consulted are shown in Table 1. The 2018 ECDC report on influenza vaccination policies was also used [23]. Regarding possible language barriers, we collect data presented in the following languages (spoken by at least of authors each): English, French, Dutch, Greek, Serbian, Italian, and Russian. We did not use online translator services such a google translator. 

A standardized form was used to collect data about vaccinations (type of vaccine, number of doses and age of administration) as well as the implementation policy (recommended or mandatory vaccinations) for adults in each of the following European countries: Albania, Austria, Belarus, Belgium, Bosnia and Herzegovina, Bulgaria, Croatia, Cyprus, Czech Republic, Denmark, Estonia, Finland, France, Germany, Greece, Hungary, Iceland, Ireland, Italy, Latvia, Liechtenstein, Lithuania, Luxembourg, Malta, Moldova, Monaco, Montenegro, Netherlands, North Macedonia, Norway, Poland, Portugal, Romania, Russia, Serbia, Slovakia, Slovenia, Spain, Sweden, Switzerland, Ukraine, and the United Kingdom. Data about vaccinations against diphtheria, tetanus, pertussis, poliomyelitis [inactivated or oral poliomyelitis vaccine (IPV or OPV, respectively)], *Haemophilus influenzae* (Hib), hepatitis A, hepatitis B, measles, mumps, rubella, varicella, herpes zoster, meningococcus group B, meningococcus group C, meningococci groups A,C,W,Y (tetravalent meningococcus vaccine A,C,W,Y), pneumococcus [7-valent, 10-valent or 13-valent pneumococcal conjugate vaccine (PCV) and 23-valent pneumococcal polysaccharide vaccine (PPV)], seasonal influenza, human papilloma virus (HPV), tuberculosis [bacillus Calmette-Guérin (BCG) vaccine], tick-born encephalitis (TBE), typhoid fever, rabies and yellow fever were collected. Vaccinations for specific high-risk groups of adults as well as catch-up vaccinations for vaccines not administered during childhood were considered. Adults were defined as persons ≥18 years old. The routine vaccination programs for children and vaccinations for healthcare personnel and travelers were not considered, nor any post-exposure vaccinations in response to outbreaks or incidental exposures to VPDs.

## 3. Results

All 42 countries had vaccination programs for adults, with a median of seven vaccinations (range: 1–18) per country. Table 2 shows vaccination programs per country and vaccine.

### 3.1. Diphtheria, Tetanus, Pertussis

Vaccination against diphtheria, tetanus and pertussis is recommended for all adults in 10 countries (Austria, Croatia, Czech Republic, where tetanus toxoid is mandatory, Finland, Germany, Greece, Latvia, Liechtenstein, Portugal and Switzerland) and it is mandatory for all adults in two (Italy and Slovenia). Vaccination against diphtheria and tetanus only, but not pertussis, is recommended in six countries (Albania, Belgium, Cyprus, France, Slovakia and Spain) and is mandatory in eight (Belarus, Bosnia and Herzegovina, Bulgaria, Moldova, Montenegro, Poland, Russia and Ukraine). In North Macedonia only the tetanus component is mandatory. In four countries (Iceland, Ireland, Serbia and the United Kingdom) the diphtheria, tetanus and pertussis vaccine is recommended only for high-risk groups. Pertussis vaccine is also recommended for specific high-risk groups in Belgium and France. No vaccinations policies are in place for diphtheria in 12 countries, tetanus in 11 countries and pertussis in 24 countries. 

In three countries some doses or some particles of the diphtheria, tetanus and pertussis vaccine are mandatory, while in some others are recommended. In Italy in particular, vaccination is mandatory at the age of 18 years but the following doses are recommended. In Czech Republic vaccination against tetanus is mandatory for all adults while vaccination against diphtheria and pertussis is recommended. In Croatia vaccination against tetanus is mandatory only for adults >60 years of age. Vaccination against diphtheria, tetanus and pertussis is recommended every ten years in Greece and for high-risk adults only in Iceland. Specifically for people >60 years of age, recommendations are in place in Austria for vaccination every five years and in France for people >65 years for vaccination every ten years. In Liechtenstein vaccination against tetanus and diphtheria is recommended at 65 years and then every 10 years and in Spain at >65 years of age. Catch-up vaccinations are implemented in Bosnia and Herzegovina, Croatia and Greece. 

Vaccination against diphtheria, tetanus and pertussis is recommended for pregnant women in 9 countries (Belgium, Czech Republic, France, Greece, Ireland, Italy, Portugal, Serbia and the United Kingdom). More specifically, Tetanus, diphtheria and acellular pertussis vaccine (Tdap) is recommended for pregnant women in all nine countries. Additional recommendations exist in Czech Republic and Greece for vaccination of mothers during the postpartum period, if vaccination was missed during pregnancy, and if breastfeeding in France. 

The most common vaccine used is Tetanus and diphtheria vaccine (Td) (16 countries, namely Albania, Belarus, Bosnia and Herzegovina, Bulgaria, Croatia, Cyprus, Estonia, Finland, Latvia, Moldova, Montenegro, Poland, Russia, Slovakia, Spain and Ukraine), followed by Tdap (fourteen countries, namely Austria, Belgium, France, Germany, Greece, Iceland, Ireland, Italy, Liechtenstein, Portugal, Serbia, Slovenia, Switzerland and the United Kingdom), Tetanus and pertussis vaccine (Tp) in Czech Republic and only Tetanus toxoid (TT) in Northern Macedonia. 

A single shot is recommended in five countries (Albania, Montenegro, Poland, Spain and Switzerland). In the remaining vaccination schemes, the intervals between the doses range from ten years (17 countries) to 20 years in France, Liechtenstein and Portugal. 

The first dose of diphtheria, tetanus, pertussis vaccine in adulthood is administered from 18 to 65 years of age. In particular, vaccination at the age of ≥18 years is recommended in 14 countries (Austria, Belgium, Bosnia and Herzegovina, Czech Republic, Germany, Greece, Italy, Moldova, Montenegro, North Macedonia, Poland, Portugal, Russia, and Ukraine), at the age of ≥25 years in 12 countries (Albania, Belarus, Bulgaria, Croatia, Cyprus, Estonia, Finland, France, Latvia, Liechtenstein, Slovenia and Switzerland), and at the age of >30 years in Serbia and Slovakia. Finally, a single dose is administered at the age of ≥65 years in Spain. After the first booster dose in adult life, the same vaccine is used in all countries but five. In particular, in France they switch from Tdap at >25 years to Td at >45 years; in Greece from Tdap at 18 years to Td every 10 years; in Portugal from Tdp at 18 years to Td at 25, 45, and 65 years; in Slovenia one booster dose includes pertussis and in Serbia from Tdap at >30 years to Td or TT only, thereafter. 

### 3.2. Poliomyelitis 

Vaccination against poliomyelitis is recommended for all adults in five countries (Austria, Croatia, France, Germany and Greece) and it is mandatory for all adults in Italy. In Iceland and Switzerland vaccination is recommended for specific risk groups every ten years (for persons at high-risk for exposure to poliomyelitis in the latter), while in the United Kingdom it is considered for pregnant women only. One dose of IPV is mandatory for refugees and asylum seekers from endemic countries upon arriving in Belgium. Catch-up vaccination is recommended in four countries (in Croatia at the age of 19 years, in France for adults >25 years, in Germany for those ≥18 years and in Greece at the age of 18 years). Booster vaccinations are recommended in Austria (at the age of ≥18 years and then every 10 years) and in France (at the age of 25 years and then every 20 years, until the age of 65 years and thereafter every ten years). All countries use the IPV. In the remaining 32 countries there are no vaccination policies against poliomyelitis specifically for adults. 

### 3.3. Haemophilus Influenzae 

Vaccination against Hib is recommended for specific risk groups in three countries (Czech Republic, France, Greece) while vaccination is mandatory for specific risk groups in Montenegro and Serbia. In particular vaccination against Hib is mandatory in Montenegro and Serbia for organ and tissue transplant recipients, patients with splenectomy and sick cell anemia, patients receiving chemotherapy or radiation therapy because of malignant tumors, and patients with other clinically established immunodeficiencies. In Serbia vaccination is also mandatory for patients with asymptomatic or symptomatic HIV infection. In particular vaccination is recommended for persons with blood diseases in France. Vaccination doses range from one to three. In France, catch-up doses are administered to high risk groups. In the remaining 37 countries there are no vaccination policies against Hib for adults.

### 3.4. Hepatitis B 

Vaccination against hepatitis B is included in adult vaccination programs in 20 countries. In particular, vaccination is recommended for all adults in Cyprus and Czech Republic in case of no vaccination during childhood, while in Austria and Greece there is a catch-up dose depending on history of vaccination, available to those over the age of 18 years. Vaccination against hepatitis B is recommended for specific risk groups in Belgium, Finland, Greece, Iceland, Italy, Latvia, Netherlands, Poland, Portugal, Serbia, Slovenia and Spain (two, three or four doses). Risk groups include people of clinical or behavioral risk, patients undergoing hemodialysis or immigrants from endemic countries. In France vaccination against hepatitis B is recommended for adults not previously vaccinated and exposed to an increased risk of contamination and specifically for persons with multiple sexual partners, injection drug users, persons receiving transfusions or blood-derived products (hemophiliacs, patients on dialysis, renal failure), candidates for organ, tissue or cell transplant, prisoners, security employees, firemen, and household members or sexual partners of persons with hepatitis B or chronic carriers of HBs antigen. Vaccination against hepatitis B is mandatory for adults, if not vaccinated already in childhood, at the age of 18–55 years in Russia, and also it is mandatory for specific groups in Bosnia and Herzegovina, Czech Republic, North Macedonia, Montenegro and Serbia (specifically for intravenous drug users, sexual partners of HBsAg positive persons, residents of institutions for mentally disabled persons, persons convicted in institutions for the criminal sanctions, home contacts of HBsAg positive persons). In the remaining 22 countries there are no vaccination policies against hepatitis B specifically for adults. 

### 3.5. Hepatitis A 

Eleven countries implement vaccinations against hepatitis A in adults. More specifically, vaccination is recommended for high-risk groups in Belgium, Bosnia and Herzegovina, Cyprus, Finland, France, Greece, Italy, Montenegro, Serbia and Spain. In particular vaccination is recommended in Belgium for employees in the food chain, in France for patients with cystic fibrosis or chronic hepatic disease, for men who have sex with men, for family members or co-habitants of a patient with hepatitis A, and in communities with poor hygiene conditions when there is a case of hepatitis A case, in Greece for susceptible persons in contact with patients with acute hepatitis A, residents and employees of closed settings for specific populations, intravenous drug users, men who have sex with men, and persons with chronic hepatic disease, in Italy for residents in regions where there are epidemics, family members or those who have close contact with people with hepatitis A, people with chronic liver disease, drug users, people working in research laboratories, and men who have sex with men, and in Spain for men who have sex with men, for illegal drug users, for patients who have a chronic liver disease such as hepatitis B or C, for patients who receive treatment with clotting factor concentrates, for people who work with animas infected with hepatitis A or in a hepatitis A research laboratory, and for people in contact with an adopted child from a country where hepatitis A is common. In Serbia in particular, vaccination is mandatory specifically for intravenous drug users, liver transplantation patients, persons working under poor hygiene conditions (e.g., in waste disposal and cemeteries) and for men who have sex with men. In Czech Republic vaccination against hepatitis A is mandatory for specific risk groups and recommended for all population if susceptible and there is no history of vaccination. In the remaining 31 countries there are no recommendations for vaccinations against hepatitis A for adults.

### 3.6. Measles, Mumps, Rubella (MMR) 

Vaccinations against MMR (one, two or three vaccine components) are included in the national vaccination programs for adults in 15 countries. All three vaccine components (MMR) are part of the national vaccinations programs in 11 countries; vaccinations against measles and rubella are included in the vaccination program in Russia, for measles only in Germany and the United Kingdom and for rubella only in Serbia. More specifically, in Russia vaccination against rubella is mandatory only for women aged 18–25 years old, while vaccination against measles is mandatory for women under the age of 35 years and it is recommended for women aged 35–55 years. In a similar vein, in Serbia vaccination against rubella is recommended for women planning a pregnancy who are unimmunized or with unknown vaccine status or seronegative; in all these cases a single dose of MMR is recommended. Additionally, in Greece, women found to be seronegative against rubella during pregnancy should be vaccinated in the post-partum period. Catch-up doses are recommended to adults depending of history of past vaccinations or in absence of evidence of immunization in Austria, Belarus, Denmark, Greece, and Italy. In Switzerland MMR is administered to adults born after 1963, in Belgium for people born after 1970, in France and in Luxembourg for people born after 1980 and in Ireland for people born after 1978. In the United Kingdom one catch-up vaccination against measles is recommended for persons born between 1997 and 2003, and in Germany to persons born after 1970 who are unvaccinated or partially vaccinated against measles. In Poland one additional dose of MMR is given by the age of 19 years to persons who did not receive the MMR vaccine at the age of ten years. Finally in Ireland the MMR vaccine is recommended for migrants coming from low-resource countries less likely to have been vaccinated and other high-risk groups and in Italy for high-risk persons with occupational risk. 

### 3.7. Varicella

Vaccination against varicella zoster virus (VZV) is recommended for high-risk groups in seven countries (Belgium, Bosnia and Herzegovina, Czech Republic, France, Greece, Switzerland and the United Kingdom) and mandatory for high-risk groups in one (Serbia). Specific recommendations are in place in Greece for catch-up vaccination of adults <26 years and for post-partum vaccination of women with no serologic proof of immunity during pregnancy. In Belgium and the United Kingdom vaccination is recommended for healthy susceptible close household contacts of immunocompromised patients. In Belgium and Serbia it is also recommended for adults with no history of chickenpox, following serologic testing, while in Greece it is recommended for all adolescents or adults <26 years old with no history of chickenpox or serologic proof of immunity. In France vaccination against VZV is recommended for women of childbearing age, especially if they plan to become pregnant, in women after a pregnancy and on effective contraception, in persons in close contact with immunocompromised patients. In Greece vaccination is specifically recommended for susceptible family members of persons at high-risk for varicella-associated serious morbidity (e.g., immunocompromised patients), persons at high risk for exposure and transmission of chickenpox (e.g., teachers, residents in closed settings, students in dormitories, military recruits), women at childbearing age that plan to become pregnant, HIV-seropositive patients with >200 CD4 cells, patients with aspleny, chronic cardiac, respiratory, hepatic or end-stage renal diseases in hemodialysis, chronic alcoholism, diabetes mellitus, and men who have sex with men. In Serbia vaccination against varicella is also mandatory for seronegative home contacts of individuals who are at high risk of severe varicella and clinically stable HIV infected patients. In Switzerland catch-up vaccination is recommended for adults <40 years and especially for women expected to be pregnant or future or young parents. Vaccination schemes include 1 to 2 doses. In the remaining 34 countries there are no vaccination recommendations against varicella specifically for adults. 

### 3.8. Herpes Zoster 

Vaccination against herpes zoster is included in adults’ vaccination programs in nine European countries. In particular, vaccination is recommended for adults aged >50 years in Austria and Czech Republic, >60 years in Germany, Greece and Spain, >65 years in France and Italy, and >70 years in the United Kingdom. An upper age limit of 74 years is defined in France only. Specific recommendations for high risk groups are in place in Czech Republic (≥18 years), Italy (50–65 years) and Serbia. No vaccination recommendations against herpes zoster exist in the remaining 33 countries.

### 3.9. Tuberculosis 

Vaccination against tuberculosis is recommended for adults in Cyprus, Ireland and Portugal. In particular, vaccination is administered only in specific high-risk groups with continuous contact with a contagious person with tuberculosis. In the remaining 39 countries there are no recommendations for vaccination against tuberculosis in adulthood.

### 3.10. Human Papilloma Virus (HPV) 

Vaccination against HPV is recommended for adults in 14 countries (Austria, Czech Republic, Denmark, France, Greece, Italy, Liechtenstein, Luxembourg, Moldova, Russia, Serbia, Switzerland, Ukraine and the United Kingdom). Vaccination is recommended for women in all the above countries. Vaccination is recommended also for men in six countries (Austria, Czech Republic, France, Italy, Liechtenstein and Russia), and in particular in France and Greece vaccination is recommended for men who have sex with men until the age of 26 years. In Greece and Serbia vaccination is also recommended for immunosuppressed persons, including HIV-seropositive persons (18–26 years old in Greece and ≥18 years in Serbia). 

Regarding HPV vaccine types, the 9-valent vaccine is recommended in Austria, Czech Republic, Italy, Luxembourg, and Switzerland, the 2-valent and 4-valent HPV vaccines in Russia, Serbia and Ukraine, the 2-valent and the 9-valent HPV vaccines in Greece, the 4-valent in Liechtenstein and the United Kingdom and all three vaccines in Denmark, France, and Moldova. The recommended doses are two in six countries (Czech Republic, Denmark, Liechtenstein, Luxembourg, Switzerland and the United Kingdom) and three in 8 countries (Austria, France, Greece, Italy, Moldova, Russia, Serbia and Ukraine). In Czech Republic a 3^rd^ dose is recommended for specific risk groups >27 years. The HPV vaccination starts from childhood and in most countries, it is continued in adulthood, while in some others it starts in adulthood. Regarding the adult population, vaccination starts at the age of 18 years and is most often completed by the age of 29. In Moldova and Russia vaccination is administered up to the age of 45 years while in Austria up to the age of 60 years. A catch-up vaccination is recommended in five countries: Austria, Denmark, Liechtenstein, Luxemburg and the United Kingdom. No recommendations for vaccination against HPV are in place in 28 countries. 

### 3.11. Seasonal Influenza 

All 42 countries have influenza vaccination policies for adults with high-risk chronic conditions. Targeted high-risk groups typically include adults with chronic pulmonary, cardiovascular and renal diseases, metabolic disorders and patients with immunosuppression. Vaccination of high-risk groups is mandatory only in Serbia and Slovakia and recommended in the remaining countries. The majority of countries (37) issued recommendations for vaccination of residents of long-term care facilities. Lastly all countries except Moldova recommend vaccination for adults of a certain age: for adults >18 years in Austria, Slovenia and Serbia; for adults ≥50 years in Ireland; for adults ≥55 years in Malta and Poland; for adults >59 years in Slovakia; for adults ≥60 years in eight countries (Albania, Germany, Greece, Hungary, Iceland, Netherlands, Russia and Ukraine); and for adults ≥65 years in 26 countries. 

Influenza vaccination is recommended to pregnant women regardless of trimester in 29 countries (Albania, Austria, Belarus, Czech Republic, Estonia, Finland, France, Greece, Hungary, Iceland, Ireland, Latvia, Liechtenstein, Lithuania, Luxembourg, Monaco, Montenegro, North Macedonia, Poland, Portugal, Romania, Russia, Serbia, Slovakia, Slovenia, Spain, Switzerland, Ukraine, United Kingdom). Influenza vaccination is recommended for healthy pregnant women in the second or third trimester and for those with chronic medical conditions regardless of trimester in Denmark, Germany, Norway, and Sweden. Recommendations for vaccination of pregnant women in the second and third trimester of pregnancy exist in Belgium, Cyprus and Italy. Two countries (Croatia and the Netherlands) recommended vaccination only for pregnant women with chronic medical conditions. Four European countries (Bulgaria, Bosnia and Herzegovina, Malta and Moldova) have no recommendations for influenza vaccination for pregnant women. 

### 3.12. Meningococcus B (MenB) 

MenB vaccine is included in the national vaccination programs for adults in six European countries (Czech Republic, France, Germany Greece, Poland and Slovenia). In particular, in Czech Republic, Men B vaccine is recommended for adults in high risk (occupational risk and people with certain medical conditions) in two doses and a booster dose is recommended for persons with a persistent risk of infection. In France, Greece and Slovenia vaccination is recommended for specific high-risk groups (in France for certain blood disorders). Specifically, in Greece and Slovenia Men B vaccination is recommended for patients with anatomic or functional aspleny, those with deficiency of specific complement fractions and those under treatment with monoclonal antibodies. In Poland Men B is recommended for adults in risk (occupational risk or residing in closed communities and immunodeficiency disorders) and recommended for vulnerable adolescents and persons over 65 years of age. Men B vaccine in addition to tetravalent meningococcus vaccine against A,C,W,Y serogroups (MCV4) is recommended for persons with certain underlying medical diseases in Germany. In the remaining 36 countries there are no recommendations for vaccination against meningococcus serogroup B for adults.

### 3.13. Meningococcus C 

Vaccination against meningococcus serogroup C is included in the vaccination schedules for adults of two European countries. In particular, in France meningococcal conjugate vaccine C is recommended as catch-up vaccination for young adults (≤24 years) who have not received previous primary vaccination as a one-dose schedule. In Poland two doses of meningococcal conjugate vaccine C are recommended in childhood with the second dose administered up to 19 years but also for adults >65 years. The remaining 40 countries have no specific vaccination recommendations for adults.

### 3.14. Meningococcus A,C,W,Y

Vaccination against meningococci serogroups A,C,W,Y with tetravalent vaccine (MCV4 and/or MPSV4) is included in the vaccination programs for adults in 14 countries. In 11 countries (Bosnia and Herzegovina, Cyprus, Czech Republic, France, Germany, Greece, Montenegro, North Macedonia, Poland, Slovenia and the United Kingdom) vaccination is recommended for specific high-risk groups (various immunodeficiency disorders including asplenia, complement deficiencies or HIV infection in advanced stage and people with occupational risk). In Serbia vaccination is mandatory for high-risk groups but also recommended for students living in university campuses and military recruits. In Russia vaccination is mandatory for adults in military service and those who live in endemic areas. For persons at continuous risk of infection, booster doses (at 3 or 5 year intervals) are recommended in Bosnia and Herzegovina, Czech Republic, France, Montenegro, North Macedonia and Serbia. Catch-up vaccination for those who missed vaccine shots in childhood or adolescence, is recommended in Greece and the United Kingdom (in the United Kingdom vaccination is performed until the age of 25 years). Meningococcal conjugate vaccine (MCV4) is recommended to all adolescents up to 18 years in Italy and in Poland for adolescents and adults >65 years of age. No recommendations for vaccination with the meningococcus A,C,W,Y vaccine exist in the remaining 28 European countries. 

### 3.15. Pneumococcal Vaccination (PPV and PCV) 

Recommendations for pneumococcal vaccine are present in 29 European countries (PPV is used as a standalone vaccine in 10 countries, PCV in five countries while 14 countries administer both vaccines). PPV is recommended as a standalone vaccine in specific adult age groups in Germany (>60 years), and Ireland, Norway and Sweden (≥65 years). In the United Kingdom PPV is recommended for adults aged 65–85 years and high-risk groups <65 years. The high-risk groups for pneumococcal disease commonly include people with chronic diseases; alcoholism; anatomic or functional asplenia; cerebrospinal fluid leakage’ inherited or acquired immunodeficiency and candidates for organ or stem cell transplantation or cochlear implants. Vaccination of high-risk groups with PPV regardless of age is specifically recommended in Bosnia and Herzegovina, Cyprus, Monaco, Montenegro and North Macedonia. Recommendations for mixed schedules of PCV followed by PPV (minimum 8 weeks apart) are in place for both high-risk groups and adults of certain age in Belgium (adults >85 and for high-risk groups 19–85 years), Denmark, Greece, Italy, Finland, Luxemburg, Russia, Slovenia and Spain (for adults ≥65 years and high-risk groups in the age 18–64 years), Iceland (for adults ≥60 years and high risk groups regardless of age), and Austria and Hungary (≥50 years and high-risk groups aged 18–49 years). In France vaccination with PCV followed by PPV is recommended only for specific high-risk groups. Mandatory vaccinations of high-risk groups aged 18–64 years along with recommended vaccination of adults ≥65 years are in place in Czech Republic (PCV only) and Serbia (PCV/PPV mixed schedule). Specifically, in Czech Republic, PCV is mandatory for people hospitalized at long-term illness wards and houses for seniors, health disability and houses with special regime. Booster vaccination with PPV is recommended in Germany (every 6 years if indicated) and in high-risk groups in France (once after 5 years), Belgium, Luxemburg, and Russia (every 5 years), Hungary (minimum two doses at intervals of 5 to 10 years), and Montenegro and Serbia (single dose after 5 years). In Slovakia PCV is recommended for adults ≥60 years and mandatory to persons residing in social care facilities. Recommendations for PCV as a standalone vaccine are in place Lithuania (for high-risk groups), Malta (for adults ≥65 years) and Poland (for adults ≥50 years). In terms of PCV vaccine, the 13-valent is used in all countries except Malta and Poland where the 10-valent vaccine is also used. No recommendations for vaccination against pneumococcal infections exist in the remaining 13 European countries.

### 3.16. Tick-borne Encephalitis (TBE) 

Vaccination against TBE is recommended for all adults in Austria, Czech Republic and Latvia; for high-risk groups in Bosnia and Herzegovina (according to epidemiological indications), Russia and Serbia (mainly for persons at occupational risk in endemic areas); for permanent residents of the island of Åland in Finland; and for adults who reside in endemic areas or for those who often go on walks in Slovenia. Most countries use three-dose schemes (one to three months interval between dose 1 and dose 2, and 5–12 months between dose 1 and dose 3). Revaccination is recommended in Austria with one dose after three years and every 5 years until the age of 60 years; for those >60 years, revaccination is recommended every three years. In Czech Republic revaccination is recommended only for adults >60 years and it is administered every three years. No recommendations for vaccination against TBE are in place in 34 countries.

### 3.17. Typhoid Fever 

Vaccination against typhoid fever is mandatory specifically for family members of *Salmonella typhi* carriers in Bosnia and Herzegovina, Montenegro and North Macedonia. The vaccination scheme consists of one dose followed by a booster dose. No recommendations for typhoid fever vaccination exist in the remaining 39 countries. 

### 3.18. Rabies 

Pre-exposure vaccination against rabies in recommended for specific high-risk groups (speleologists and persons at occupational risk) in Czech Republic. In the United Kingdom vaccination against rabies is recommended for individuals at continuous and frequent risk of exposure to rabies virus. Vaccination is mandatory for specific high-risk groups in Bosnia and Herzegovina, Montenegro, North Macedonia, and Serbia. High-risk groups include persons professionally exposed to rabies virus such as laboratory workers directly exposed to rabies virus, veterinarians, veterinary hygienists, zoo hygienists, hunters, foresters and animal preparers, furriers, and persons who professionally come into contact with bats. The vaccination scheme consists of two doses (7 days apart) in Bosnia and Herzegovina, followed by a booster dose administered periodically, depending on the level of serum antibodies of vaccine recipient; or three doses administered according to the 0, 7 and 21 day scheme, in Czech Republic, Montenegro, North Macedonia and Serbia, followed by a booster dose one dose later in Montenegro and revaccination after 2–5 years in Czech Republic. No recommendations for rabies vaccination are in place in in the rest, 36, countries. 

### 3.19. Yellow Fever 

Vaccination against yellow fever in mandatory for residents of French Guiana. No recommendations exist in the rest of European countries.

### 3.20. Mandatory Vaccinations for Adults in Europe

Mandatory vaccination policies for adults are implemented in the following 17 countries: Belarus, Belgium, Bosnia and Herzegovina, Bulgaria, Croatia, Czech Republic, France, Italy, Moldova, Montenegro, North Macedonia, Poland, Russia, Serbia, Slovakia, Slovenia, and Ukraine. Mandatory policies concern mainly vaccinations against diphtheria, tetanus and hepatitis B. 

## 4. Discussion

We studied the current vaccination programs for adults in Europe. All European countries have national vaccination programs for adults. However, there are significant variations between European vaccination programs in terms of number of vaccines, vaccination schedules, target groups, and regulatory frame of implementation (voluntary or mandatory vaccinations). Similar variations have been found between European vaccination programs for children and for healthcare personnel [24]. Differences between countries on incidence and epidemiological trends of specific VPDs and vaccination coverage rates in children may partially explain these differences. Cost issues, capacities for vaccine reimbursement, different criteria for the introduction of vaccines in national vaccination programs, and lack of licensed vaccines in a country also have an impact on vaccination policies. 

All European countries have influenza vaccination policies for high-risk groups, including older adults, and most of them for residents of long-term care facilities. This is attributed to the decade-long European Council recommendation to improve influenza vaccine uptake among older age and high-risk groups [25]. Similarly, the overwhelming majority of European countries have issued influenza vaccination recommendations specifically for pregnant women. This is in accordance with the WHO consideration of pregnant women as a high-risk priority group for influenza vaccination [26]. 

Beyond influenza vaccination, most European countries have vaccination policies against diphtheria, tetanus and pneumococcus specifically for adults. This is attributed to the need for booster shots against diphtheria and tetanus in order to achieve and sustain immunity during adulthood. The high morbidity and mortality burden of invasive pneumococcal disease in older age and high-risk groups explains the implementation of pneumococcal vaccination policies for these groups in several European countries [27,28]. 

Furthermore, our study revealed significant gaps in vaccination policies for adults. In particular, less than half of European countries have vaccination policies against hepatitis B and pertussis. Vaccination against pertussis is of particular importance given the shift in the age distribution of pertussis towards adolescents and young adults in recent years, especially in countries where acellular vaccines replaced whole-cell pertussis vaccines for primary vaccine series [29]. As of 2019, only nine European countries have vaccination policies against pertussis for pregnant women, despite the fact that vaccination during pregnancy is likely to be the most cost-effective strategy for conferring immunity against pertussis in infants too young to be vaccinated [27]. 

Less than one third of European countries have vaccination policies against measles and rubella and only one fourth of them have vaccination policies against mumps and hepatitis A specifically for adults, despite the large epidemics of the respective VPDs the preceding years in Europe [4,5,6]. Several countries may count on their routine pediatric vaccination programs to protect adults. However the several thousands of notified cases of measles and tens of deaths among young adults the last years indicate that this was inadequate [6]. The implementation of vaccination policies against measles, rubella and hepatitis B during adulthood is in accordance with the WHO European Vaccine Action Plan 2015–2020 goals for elimination of measles and rubella and control of hepatitis B [30]. 

Few European countries have vaccination policies against varicella for adults specifically, despite the high morbidity of this disease in this age group and specifically in high-risk groups. Similarly, most countries have no vaccination policies against herpes zoster, which may be attributed to concerns over the moderate effectiveness of the herpes-zoster vaccine [31]. Vaccination policies against HPV were in place in 14 countries, and in most of them act complimentary to HPV vaccination policies targeting adolescents. Few countries implement vaccinations against invasive meningococcal disease for adults and these concern almost exclusively high-risk persons. Lastly, vaccination policies against TBE, rabies, tuberculosis, typhoid fever, and yellow fever exist in few countries based on national or local epidemiology data or high-risk conditions. 

As of 2019, mandatory vaccination policies for adults exist in 17 European counties, mainly against diphtheria, tetanus and hepatitis B. Mandatory vaccination policies for children against several VPDs were recently adopted in Italy, France and Germany, with very good preliminary results [10,32,33]. Mandatory vaccination policies for healthcare personnel are also in place in several European countries [24]. It is likely that mandatory vaccination policies will be increasingly implemented, considering the recent devastating measles epidemics in Europe and the increasing vaccine hesitancy [6,34]. Beyond comprehensive vaccination policies, achievement of high and sustainable vaccination policies requires the successful implementation of additional policy elements, including funding, easy access to vaccines and vaccination services, good monitoring of vaccination rates, and reminding systems [11,20,35,36,37].

The main strength of our study is the participation of almost all European countries and the fact that we used the official public health websites for data retrieval. Our study can contribute to the establishment of a common vaccination program for adults across Europe. The definitions of ‘recommended’ and ‘mandatory’ may vary between countries, which constitutes a potential limitation. Moreover, we did not consider issues of re-imbursement of vaccines and vaccinations.

## 5. Conclusions 

In conclusion, there are considerable differences between vaccination programs for adults in Europe, in terms of number of vaccines, timing of vaccinations, target groups, and regulatory frame of implementation. All European countries have influenza vaccination policies for high-risk groups, including older adults, and most of them for pregnant women and residents of long-term care facilities. A core vaccination program against diphtheria, tetanus and pneumococcus is in place in most European countries. However, there are significant gaps in vaccination policies especially against measles, mumps, rubella, varicella, pertussis, hepatitis B, and hepatitis A. As of 2019, 17 European countries implement mandatory vaccination policies for adults. The national vaccination programs for adults should be strengthened, considering the current epidemiological trends of VPDs and the changes in the demographic structure of the European population [35]. A consensus-based vaccination policy at the European level is needed. 

## Figures and Tables

**Table 1:** Consulted official public health websites.

Country	Websites
All countries (ECDC)	https://vaccine-schedule.ecdc.europa.eu/
All countries (WHO)	http://apps.who.int/immunization_monitoring/globalsummary/schedules
Serbia	https://www.paragraf.rs/propisi/pravilnik_o_imunizaciji_i_nacinu_zastite_lekovima.html http://www.batut.org.rs/download/publikacije/SMU%20imuinizacija.pdf
Bosnia and Herzegovina	http://mz.ks.gov.ba/sites/mz.ks.gov.ba/files/naredba_za_2019-_broj026.pdf https://vakcine.ba/kalendari-imunizacije-2/kalendar-imunizacije-rs-2019/ https://www.zzjzfbih.ba/imunizacija/
North Macedonia	http://zdravstvo.gov.mk/wp-content/uploads/2019/08/Programa-za-zadolzhitelna-imunizatsija-na-naselenieto-vo-RM-za-2019-godina.pdf
Montenegro	http://www.mzdravlja.gov.me/biblioteka/dokument http://www.dzpg.me/kalendar-obaveznih-imunizacija/
Poland	https://gis.gov.pl/wp-content/uploads/2018/01/akt.pdf
Czech Republic	https://www.vakcinace.eu/data/files/downloads/ockovaci_kalendar_dospsdatem.pdf http://www.szu.cz/tema/vakciny/ockovaci-kalendar-v-cr
United Kingdom	https://www.gov.uk/government/publications/menacwy-programme-information-for-healthcare-professionals
France	https://vaccination-info-service.fr/var/vis/storage/original/application/download/calendrier-vaccinal-2019.pdf https://vaccination-info-service.fr/La-vaccination-au-cours-de-la-vie/Adultes-20-a-64-ans
Belarus	http://minzdrav.gov.by/ru/dlya-belorusskikh-grazhdan/vaktsinatsiya/natsionalnyy-kalendar-privivok.php
Russia	https://www.rosminzdrav.ru/poleznye-resursy/klinicheskie-rekomendatsii-po-vaktsinoprofilaktike-pnevmokokkovoy-infektsii https://www.rospotrebnadzor.ru/deyatelnost/epidemiological-surveillance/?ELEMENT_ID=5575
Ukraine	https://moz.gov.ua/dokumenti
Moldova	https://msmps.gov.md/en
Belgium	https://www.laatjevaccineren.be/vaccinaties-voor-volwassenen-19-64-jaar https://www.vaccination-info.be/adultes/
Switzerland	https://www.infovac.ch/docs/public/fs/plan-de-vaccination-2019.pdf
Italy	http://www.salute.gov.it/imgs/C_17_pagineAree_4829_listaFile_itemName_0_file.pdf
Greece	http://www.isth.gr/images/uploads/files/emvoliasmos2018-2019.pdf

ECDC: European Centre for Disease Prevention and Control; WHO: World Health Organization.

**Table 2:** National vaccination policies for adults in Europe by vaccine and by country, 2019.

Country	D	T	P	Polio	Hib	HepB	HepA	Me	Mu	R	VZV	HZ	BCG	HPV	Flu	MenΒ	MenC	MCV4	PPV	PCV	TBE	TF	Rabies	YF
**Albania**	R	R	nMnR	nMnR	nMnR	nMnR	nMnR	nMnR	nMnR	nMnR	nMnR	nMnR	nMnR	nMnR	R/spR	nMnR	nMnR	nMnR	nMnR	nMnR	nMnR	nMnR	nMnR	nMnR
**Austria**	R	R	R	R	nMnR	R	nMnR	R	R	R	nMnR	R	nMnR	R	R/spR	nMnR	nMnR	nMnR	R/spR	R/spR	R	nMnR	nMnR	nMnR
**Belarus**	M	M	nMnR	nMnR	nMnR	nMnR	nMnR	R	R	R	nMnR	nMnR	nMnR	nMnR	R/spR	nMnR	nMnR	nMnR	nMnR	nMnR	nMnR	nMnR	nMnR	nMnR
**Belgium**	R/spR	R/spR	spR	spM	nMnR	spR	spR	R	R	R	R/SpR	nMnR	nMnR	nMnR	R/spR	nMnR	nMnR	nMnR	R/spR	R/spR	nMnR	nMnR	nMnR	nMnR
**Bosnia Herzegovina**	M	M	nMnR	nMnR	nMnR	spM	spR	nMnR	nMnR	nMnR	spR	nMnR	nMnR	nMnR	R/spR	nMnR	nMnR	spR	spR	nMnR	spR	spM	spM	nMnR
**Bulgaria**	M	M	nMnR	nMnR	nMnR	nMnR	nMnR	nMnR	nMnR	nMnR	nMnR	nMnR	nMnR	nMnR	R/spR	nMnR	nMnR	nMnR	nMnR	nMnR	nMnR	nMnR	nMnR	nMnR
**Croatia**	R	M/R	R	R	nMnR	nMnR	nMnR	nMnR	nMnR	nMnR	nMnR	nMnR	nMnR	nMnR	R/spR	nMnR	nMnR	nMnR	nMnR	nMnR	nMnR	nMnR	nMnR	nMnR
**Cyprus**	R	R	nMnR	nMnR	nMnR	R	spR	nMnR	nMnR	nMnR	nMnR	nMnR	spR	nMnR	R/spR	nMnR	nMnR	spR	R/spR	nMnR	nMnR	nMnR	nMnR	nMnR
**Czech Republic**	R/spR	M/R/spR	R/spR	nMnR	spR	spM/R	R/spM	nMnR	nMnR	nMnR	spR	R/spR	nMnR	R/spR	R/spR	spR	nMnR	spR	nMnR	spM/R	R	nMnR	spR	nMnR
**Denmark**	nMnR	nMnR	nMnR	nMnR	nMnR	nMnR	nMnR	R	R	R	nMnR	nMnR	nMnR	spR	R/spR	nMnR	nMnR	nMnR	R/spR	R/spR	nMnR	nMnR	nMnR	nMnR
**Estonia**	nMnR	nMnR	nMnR	nMnR	nMnR	nMnR	nMnR	nMnR	nMnR	nMnR	nMnR	nMnR	nMnR	nMnR	R/spR	nMnR	nMnR	nMnR	nMnR	nMnR	nMnR	nMnR	nMnR	nMnR
**Finland**	R	R	R	nMnR	nMnR	spR	spR	nMnR	nMnR	nMnR	nMnR	nMnR	nMnR	nMnR	R/spR	nMnR	nMnR	nMnR	R/spR	R/spR	spR	nMnR	nMnR	nMnR
**France**	R/spR	R/spR	spR	R	spR	nMnR	spR	R	R	R	spR	R	nMnR	spR	R/spR	spR	R	spR	spR	spR	nMnR	nMnR	nMnR	spM
**Germany**	R	R	R	R	nMnR	nMnR	nMnR	R	nMnR	nMnR	nMnR	R	nMnR	nMnR	R/spR	spR	nMnR	spR	R	nMnR	nMnR	nMnR	nMnR	nMnR
**Greece**	R/spR	R/spR	R/spR	R	spR	spR/R	spR	R/spR	R/spR	R/spR	R/spR	R	nMnR	spR	R/spR	spR	nMnR	spR	R/spR	R/spR	nMnR	nMnR	nMnR	nMnR
**Hungary**	nMnR	nMnR	nMnR	nMnR	nMnR	nMnR	nMnR	nMnR	nMnR	nMnR	nMnR	nMnR	nMnR	nMnR	R/spR	nMnR	nMnR	nMnR	R/spR	R/spR	nMnR	nMnR	nMnR	nMnR
**Iceland**	spR	spR	spR	spR	nMnR	spR	nMnR	nMnR	nMnR	nMnR	nMnR	nMnR	nMnR	nMnR	R/spR	nMnR	nMnR	nMnR	R/spR	R/spR	nMnR	nMnR	nMnR	nMnR
**Ireland**	spR	spR	spR	nMnR	nMnR	nMnR	nMnR	R/spR	R/spR	R/spR	nMnR	nMnR	spR	nMnR	R/spR	nMnR	nMnR	nMnR	R	nMnR	nMnR	nMnR	nMnR	nMnR
**Italy**	M/R/spR	M/R/spR	M/R/spR	M	nMnR	spR	spR	R/spR	R/spR	R/spR	nMnR	R/spR	nMnR	R	R/spR	nMnR	nMnR	R	R/spR	R/spR	nMnR	nMnR	nMnR	nMnR
**Latvia**	R/spR	R/spR	R	nMnR	nMnR	spR	nMnR	nMnR	nMnR	nMnR	nMnR	nMnR	nMnR	nMnR	R/spR	nMnR	nMnR	nMnR	nMnR	nMnR	R	nMnR	nMnR	nMnR
**Liechtenstein**	R	R	R	nMnR	nMnR	nMnR	nMnR	nMnR	nMnR	nMnR	nMnR	nMnR	nMnR	R	R/spR	nMnR	nMnR	nMnR	nMnR	nMnR	nMnR	nMnR	nMnR	nMnR
**Lithuania**	nMnR	nMnR	nMnR	nMnR	nMnR	nMnR	nMnR	nMnR	nMnR	nMnR	nMnR	nMnR	nMnR	nMnR	R/spR	nMnR	nMnR	nMnR	nMnR	spR	nMnR	nMnR	nMnR	nMnR
**Luxembourg**	nMnR	nMnR	nMnR	nMnR	nMnR	nMnR	nMnR	R	R	R	nMnR	nMnR	nMnR	spR	R/spR	nMnR	nMnR	nMnR	R/spR	R/spR	nMnR	nMnR	nMnR	nMnR
**Malta**	nMnR	nMnR	nMnR	nMnR	nMnR	nMnR	nMnR	nMnR	nMnR	nMnR	nMnR	nMnR	nMnR	nMnR	R/spR	nMnR	nMnR	nMnR	nMnR	R	nMnR	nMnR	nMnR	nMnR
**Moldova**	M	M	nMnR	nMnR	nMnR	nMnR	nMnR	nMnR	nMnR	nMnR	nMnR	nMnR	nMnR	spR	spR	nMnR	nMnR	nMnR	nMnR	nMnR	nMnR	nMnR	nMnR	nMnR
**Monaco**	nMnR	nMnR	nMnR	nMnR	nMnR	nMnR	nMnR	nMnR	nMnR	nMnR	nMnR	nMnR	nMnR	nMnR	R/spR	nMnR	nMnR	nMnR	spR	nMnR	nMnR	nMnR	nMnR	nMnR
**Montenegro**	M	M	nMnR	nMnR	spM	spM	spR	nMnR	nMnR	nMnR	nMnR	nMnR	nMnR	nMnR	R/spR	nMnR	nMnR	spR	spR	nMnR	nMnR	spM	spM	nMnR
**Netherlands**	nMnR	nMnR	nMnR	nMnR	nMnR	spR	nMnR	nMnR	nMnR	nMnR	nMnR	nMnR	nMnR	nMnR	R/spR	nMnR	nMnR	nMnR	nMnR	nMnR	nMnR	nMnR	nMnR	nMnR
**North** **Macedonia**	nMnR	M	nMnR	nMnR	nMnR	spM	nMnR	nMnR	nMnR	nMnR	nMnR	nMnR	nMnR	nMnR	R/spR	nMnR	nMnR	spR	spR	nMnR	nMnR	spM	spM	nMnR
**Norway**	nMnR	nMnR	nMnR	nMnR	nMnR	nMnR	nMnR	nMnR	nMnR	nMnR	nMnR	nMnR	nMnR	nMnR	R/spR	nMnR	nMnR	nMnR	R	nMnR	nMnR	nMnR	nMnR	nMnR
**Poland**	M	M	nMnR	nMnR	nMnR	spR	nMnR	R	R	R	nMnR	nMnR	nMnR	nMnR	R/spR	spR	R	R/spR	nMnR	R	nMnR	nMnR	nMnR	nMnR
**Portugal**	R/spR	R/spR	R/spR	nMnR	nMnR	spR	nMnR	nMnR	nMnR	nMnR	nMnR	nMnR	spR	nMnR	R/spR	nMnR	nMnR	nMnR	nMnR	nMnR	nMnR	nMnR	nMnR	nMnR
**Romania**	nMnR	nMnR	nMnR	nMnR	nMnR	nMnR	nMnR	nMnR	nMnR	nMnR	nMnR	nMnR	nMnR	nMnR	R/spR	nMnR	nMnR	nMnR	nMnR	nMnR	nMnR	nMnR	nMnR	nMnR
**Russia**	M	M	nMnR	nMnR	nMnR	M	nMnR	spM/spR	nMnR	spM	nMnR	nMnR	nMnR	R	R/spR	nMnR	nMnR	spM	R/spR	R/spR	spR	nMnR	nMnR	nMnR
**Serbia**	spR	spR	spR	nMnR	spM	spM/spR	spM/spR	nMnR	nMnR	spR	R/spM	spR	nMnR	spR	R/spM	nMnR	nMnR	spR/spM	spM/R	spM/R	spR	nMnR	spM	nMnR
**Slovakia**	R	R	nMnR	nMnR	nMnR	nMnR	nMnR	nMnR	nMnR	nMnR	nMnR	nMnR	nMnR	nMnR	R/spM	nMnR	nMnR	nMnR	nMnR	R/spM	nMnR	nMnR	nMnR	nMnR
**Slovenia**	M	M	M	nMnR	nMnR	spR	nMnR	nMnR	nMnR	nMnR	nMnR	nMnR	nMnR	nMnR	R/spR	spR	nMnR	spR	R/spR	R/spR	spR	nMnR	nMnR	nMnR
**Spain**	R	R	nMnR	nMnR	nMnR	spR	spR	nMnR	nMnR	nMnR	nMnR	R	nMnR	nMnR	R/spR	nMnR	nMnR	nMnR	R/spR	R/spR	nMnR	nMnR	nMnR	nMnR
**Sweden**	nMnR	nMnR	nMnR	nMnR	nMnR	nMnR	nMnR	nMnR	nMnR	nMnR	nMnR	nMnR	nMnR	nMnR	R/spR	nMnR	nMnR	nMnR	R	nMnR	nMnR	nMnR	nMnR	nMnR
**Switzerland**	R	R	R	spR	nMnR	nMnR	nMnR	R	R	R	spR	nMnR	nMnR	spR	R/spR	nMnR	nMnR	nMnR	nMnR	nMnR	nMnR	nMnR	nMnR	nMnR
**Ukraine**	M	M	nMnR	nMnR	nMnR	nMnR	nMnR	nMnR	nMnR	nMnR	nMnR	nMnR	nMnR	spR	R/spR	nMnR	nMnR	nMnR	nMnR	nMnR	nMnR	nMnR	nMnR	nMnR
**United Kingdom**	spR	spR	spR	spR	nMnR	nMnR	nMnR	R	nMnR	nMnR	spR	R	nMnR	spR	R/spR	nMnR	nMnR	spR/R	R/spR	nMnR	nMnR	nMnR	spR	nMnR

D: diphtheria; T: tetanus; P: pertussis; Polio: poliomyelitis; Hib: *Haemophilus influenzae*; Hep B: hepatitis B; Hep A: hepatitis A; Me: measles; Mu: mumps; R: rubella; VZV: varicella-zoster virus; HZ: herpes zoster; BCG: bacillus Calmette-Guérin vaccine; HPV: human papilloma virus; Flu: influenza; MenB: meningococcus B; MenC: meningococcus C; MCV4: meningococci A,C,W,Y; PPV: pneumococcal polysaccharide vaccine; PCV: pneumococcal conjugate vaccine; TBE: tick-born encephalitis; TF: typhoid fever; YF: yellow fever; R: recommended for all adults; spR: recommended for specific groups; M: mandatory for all adults; spM: mandatory for specific groups; nMnR: not mandatory-not recommended.
